# Delirium: more than a marker of baseline vulnerability

**DOI:** 10.1093/ageing/afag139

**Published:** 2026-05-19

**Authors:** Rose S Penfold, Alasdair M J MacLullich

**Affiliations:** Usher Institute, School of Population Health Sciences, University of Edinburgh, Edinburgh, EH16 4UX, UK; Usher Institute, School of Population Health Sciences, University of Edinburgh, Edinburgh, EH16 4UX, UK

## Key Points

- Editorial to accompany: Frequent in Dementia, Deadliest Without It: Delirium and Mortality in Hospitalised Older Adults.
- Delirium is a strong prognostic marker for short-term mortality.
- The relative association between delirium and 90-day mortality was greatest in people without dementia.
- Delirium is not merely an ‘epiphenomenon’ of underlying vulnerability, but an independent prognostic marker.
- Screen all older adult emergency admissions routinely for delirium and treat delirium as a clinical red flag.

Delirium has long been regarded as a mere epiphenomenon arising from baseline vulnerability interacting with acute illness. A common view is that it is an inconvenient and distressing but essentially transient and benign syndrome, with any association with adverse outcomes explained by underlying frailty or dementia. Yet recent research has begun to challenge that assumption. Across several cohorts, delirium is emerging as an important prognostic signal, independent of chronic markers of baseline morbidity, that identifies patients at markedly increased risk of death or future dementia. Whatever the underlying mechanisms, delirium is increasingly best understood not only as an acute syndrome requiring urgent management, but also as a marker of future ill health, including increased risks of mortality, institutionalisation and dementia after discharge [1].

The international multicentre cohort study by Umoh *et al.* [2] addresses a clinically important question: does delirium confer the same prognostic information in people with and without pre-existing cognitive impairment? Put another way, is delirium simply a marker of baseline vulnerability, or an independent prognostic marker across the spectrum of baseline cognition?

## What this study adds

Umoh *et al.* studied 2556 patients across 43 hospitals in five countries (Brazil, Angola, Chile, Colombia and Portugal). Delirium was common, occurring in 957 (37%) participants, and—as expected—more frequently in those with baseline cognitive impairment [2]. Using the informant-rated Clinical Dementia Rating (CDR) to stage pre-admission cognition, delirium occurred in 126/765 (16%) of those with no dementia (CDR 0) and in 357/465 (77%) of those with moderate–severe dementia (CDR 2–3). [3] This steep gradient is consistent with extensive existing evidence that cognitive impairment is one of the strongest risk factors for delirium.

The key finding, however, is the interaction of delirium with baseline cognition. Delirium was associated with substantially higher 90-day mortality overall [adjusted hazard ratio (HR) 3.45], but the strongest relative association was actually in those without baseline dementia (HR 4.40). The association was weakest in those with moderate–severe dementia (HR 2.22). In other words, delirium was most common in people with dementia, but its mortality signal was strongest when it occurred in those without known dementia.

## Relative versus absolute risk

Attenuation of the *relative* mortality hazard in people with moderate–severe dementia does not mean that delirium is ‘less serious’ in this group. When baseline mortality is high, proportional effects can appear smaller even when absolute risks remain substantial. The cumulative mortality differences illustrate this: delirium roughly quadrupled 90-day mortality in those without dementia (54% vs 15%) but still doubled mortality in those with moderate–severe dementia (36% vs 17%). Delirium is therefore a high-risk mortality signal with or without dementia. However, this study suggests it may be most prognostically discriminating in those without dementia: the group in whom delirium may be least expected, and therefore most likely to be missed unless clinicians look for it systematically.

## How does this fit with earlier evidence?

The question of ‘who loses most’ after delirium has been explored previously. In the DELPHIC population-based cohort, Tsui *et al.* showed that higher baseline cognition was associated with lower delirium risk , but if delirium occurred, it was often more severe and associated with greater future cognitive decline [4]. In a large acute general medicine cohort with long-term follow-up, Gan *et al.* reported that delirium was associated with prolonged admission, greater discharge care needs and increased mortality, with relatively more adverse outcomes in younger older adults (aged 65–74 years) without comorbid dementia [5].

In a population-based cohort of 23 558 older adults without pre-existing dementia, we observed a similar pattern: delirium was associated with higher 30-day mortality risk across the spectrum of long-term conditions [6]. Furthermore, we found that delirium was consistently associated with higher incident dementia risk, and the strongest relative association was in those with no or few long-term conditions.

Taken together, the emerging signal across these studies is coherent: delirium appears to be the strongest prognostic marker of subsequent adverse outcomes in people with fewer pre-existing vulnerabilities.

## Why might delirium be a stronger signal in people without dementia?

Several mechanisms may contribute and these are not mutually exclusive.

- **Marker of acute illness severity:** in people without cognitive impairment, delirium may reflect a larger physiological insult, making it a more specific marker of severe acute illness.
- **Latent vulnerability:** delirium may be the first overt manifestation of reduced brain resilience due to subclinical neurodegeneration, cerebrovascular small vessel disease, genetic susceptibility or other susceptibility not captured by routine clinical measures [7, 8].
- **Competing risks and ceiling effects:** in moderate–severe dementia, high baseline mortality risk may reduce the observable proportional increment attributable to delirium, even if absolute mortality risks remain substantial.

## Implications for practice: routine delirium screening and baseline cognitive assessment

These findings have several important implications for clinical practice. Firstly, they support rigorous routine delirium assessment on hospital admission for all acutely hospitalised older adults, not only those with known dementia. This aligns with NHS England GIRFT standards recommending assessment using the 4AT for all emergency admissions aged over 65, SIGN guidelines and APA recommendations for structured assessment in patients with delirium or at risk of delirium [9–11]. However, screening must trigger action. A positive screen should prompt urgent clinical assessment, a rapid review for potential precipitants, and a systematic approach to preventing further harm.

Secondly, this study highlights the importance of establishing pre-admission cognition. This not only underpins delirium diagnosis (distinguishing acute change from chronic impairment), but improves prognostication, discharge planning and communication with patients and families. The 4AT can support both delirium detection and baseline cognitive ascertainment in routine care, and our recent *Age and Ageing* paper suggests that abnormal 4AT scores may also identify patients who warrant cognitive follow-up even when delirium is not diagnosed [12].

Finally, Umoh *et al.* report higher rates of in-hospital complications among those with delirium, including infection, functional decline and longer length of stay [2]. These are plausible pathways linking delirium to short-term mortality and are targets for high-quality acute geriatric care, including early mobilisation, hydration and nutrition support, pain control, medication rationalisation and prevention of avoidable harm.

## Conclusion

This international multicentre cohort study reinforces that delirium is a strong prognostic marker for short-term mortality in older adults. Although delirium occurred most frequently in people with dementia, its relative association with 90-day mortality was greatest in those *without* dementia. Across health systems and continents, across different approaches to delirium assessment, and across outcomes, the signal is clear: delirium is not merely an ‘epiphenomenon’ of underlying vulnerability, but an independent prognostic marker across the spectrum of baseline health. For clinicians, the implications are: screen all older adult emergency admissions routinely for delirium, document baseline cognition at the same time, and treat delirium as a clinical red flag that should trigger urgent further clinical assessment and action.
